# SARS-CoV-2 Contamination of Ambulance Surfaces and Effectiveness of Routine Decontamination Procedure: A Classic Hygiene Lesson for A Novel Pathogen

**DOI:** 10.3390/ijerph192013646

**Published:** 2022-10-21

**Authors:** Tatjana Baldovin, Irene Amoruso, Matteo Paganini, Camilla Marcato, Riccardo Boscolo Cegion, Andrea Favaro, Chiara Bertoncello, Marco Fonzo, Vincenzo Baldo

**Affiliations:** 1 Laboratory of Hygiene and Applied Microbiology, Hygiene and Public Health Unit, Department of Cardiac, Thoracic, Vascular Sciences and Public Health, University of Padova, 35131 Padova, Italy; 2 Department of Biomedical Sciences, University of Padova, 35121 Padova, Italy; 3 School of Hygiene and Preventive Medicine, Department of Cardiac, Thoracic, Vascular Sciences and Public Health, University of Padova, 35131 Padova, Italy; 4 Department of Emergency Medical Services, San Bassiano Hospital, ULSS7 Pedemontana, 36061 Bassano del Grappa, Italy

**Keywords:** emergency medical services (EMS), prehospital emergency medicine, COVID-19, ambulance, healthcare-associated infection (HAI), medical-transport-associated infection (MTAI)

## Abstract

The efficacy of standard operating procedures (SOPs) for the decontamination of ambulances against SARS-CoV-2 has been debated. In Italy, the differential use of ambulances was implemented by regional health authorities, with selected vehicles being used exclusively for transporting COVID-19 patients. We investigated the presence of SARS-CoV-2 on high-touch surfaces in ambulances to assess contamination dynamics and the effectiveness of decontamination SOPs. Four high-touch surfaces were sampled before and after decontamination (T0; T1). The gloves of the EMS crew chief were also sampled. RNA extraction was performed with a commercial kit, followed by RT-qPCR molecular detection of SARS-CoV-2. A total of 11 transports were considered. Seven transports had at least one positive sample; all were related to a COVID-19 patient. Three of the negative transports had dealt with COVID-19 case, and one had dealt with a COVID-19-negative patient. One door handle and one oxygen knob were positive at T0, with negative T1 swabs. The monitors were positive in 5 transports at T0, yet they were never positive at T1. Three stretcher handles tested positive at T0, and two of them also at T1, possibly having bypassed decontamination during personnel dismounting. Gloves were contaminated in five transports, in which 1 to 3 additional samples (monitor, knob, stretcher) resulted as positive. Overall, the efficacy of decontamination SOPs was confirmed under the unprecedented conditions of the COVID-19 emergency. However, the importance of correct hand-hygiene and glove-disposal should be further emphasized through the dedicated training of EMS personnel.

## 1. Introduction

Shortly after the onset of the ongoing coronavirus pandemic, concerns were raised about the efficacy of standard operating procedures (SOP) for ambulance decontamination operated by emergency medical services (EMS) crew, following the transport of both COVID-19 patients and persons under investigation (PUI). Given the degree of evidence-based uncertainty and the unprecedented burden on healthcare systems, a possible decline in the effectiveness of decontamination SOPs was hypothesized.

Prepandemic literature on the bacterial contamination of ambulances dates back to the early 2000s (e.g., [1,2]). Later studies also investigated the role of multidrug resistant strains on emergency vehicles (e.g., [3,4]). More recently, a review discussed the role of microbial pathogens in the ambulance environment, stressing how EMS personnel do not regularly comply with hygiene practices, and that patients are potentially affected by healthcare-associated infections (HAIs) as a direct result of ambulance exposure. The latter subset is referred to as medical-transport-associated infection (MTAI) [5].

In addition, considering that EMS crews were at the forefront of the fight against COVID-19, tiredness, fatigue, and a possible lack of time and resources were recognized as additional risk factors. Moreover, insufficient compliance on behalf of EMS crews with decontamination SOPs had been reported even before the pandemic [6]. The study reported on how the contamination of cabin surfaces exceeded the levels recommended for other healthcare settings, stressing the need to establish a standardized set of guidelines and policies to be shared among EMS crews.

Criticisms concerning the lack of adequate training programs for ambulance personnel were also emphasized [7]. Indeed, poorly performed decontamination techniques, including complementary measures, such as hand hygiene and the decontamination of surfaces, were previously described as potential risk factors for the infection both of transported patients and EMS crew [6,8].

In the pandemic scenario, the prompt implementation of prevention and control measures against SARS-CoV-2 resulted in being fundamental. Possible transmission mechanisms for SARS-CoV-2 include the inhalation of contaminated carriers (i.e., infected respiratory droplets or aerosol particles); the deposition of such carriers onto mucous membranes; and contact of mucous membranes with contaminated hands or environmental surfaces and objects [9]. Shifting to the EMS framework, the contamination of environmental surfaces by SARS-CoV-2 appears to be critical. Primarily, regularly touched surfaces represent the highest risk for viral transmission [10]. Previously published studies showed that SARS-CoV-2 survival on surfaces can last a considerable timespan (i.e., up to 21 days on steel and glass) [11] although environmental temperatures are capable of significantly influencing viral persistence [12]. Consistently, the frequent cleaning and disinfection of surfaces are recommended [7].

To the authors’ knowledge, only a few works are available in the literature that discuss the possible viral transmission pathways through contaminated surfaces in ambulances [13,14,15]. The present study thus aimed to investigate the potential risk of infection in ambulances through dedicated surface sampling methods that consider the on-board procedures and specific actions of the EMS crew during the transport of COVID-19 cases or PUIs. The study design is that of a pre-/post investigation since the presence of SARS-CoV-2 RNA on selected ambulance surfaces was evaluated at the end of selected EMS transports, both before and after the decontamination procedures.

## 2. Materials and Methods

The present investigation was carried out in the Veneto Region (NE Italy) during the first stages of the COVID-19 pandemic (10 March 2020–30 April 2020). Two EMS bases were involved in the study: the Italian Red Cross Committee of Selvazzano Dentro (province of Padova) and the Emergency Department of Bassano del Grappa (province of Vicenza). Convenience sampling of COVID-19 EMS transports was performed by adopting a set of real-time decisional processes from information provided by the EMS Operations Center. One heart-failure transport was also considered, serving as an experimental blank. Transported patients were not directly involved in the study, and their sensitive personal data were not accessible, nor needed, by the investigators. Results of SARS-CoV-2 molecular testing performed upon hospital admission were also recovered for each patient for experimental completeness.

For each EMS base, one crew that expressed a willingness to participate in an internal quality improvement initiative attended a 1-hour training on COVID-19 surface sampling techniques, held by the Laboratory of Hygiene and Applied Microbiology (LIMA) of the University of Padova. The training included a theoretical webinar, a dedicated surface-swabbing video tutorial and a conclusive practical session. The experimental details and the precise scope of the study were divulged to neither the EMS trainees nor the decontamination technicians, so as to adopt blinded-experiment precautions and to reduce the risk of experimental bias arising from the awareness and knowledge of the field investigators.

As a reference the WHO’s environmental surface sampling protocol for SARS-CoV-2 was adopted [16]. Four high-touch ambulance surfaces were selected for sampling: the stretcher handrail (S), the sliding-door handle (H), the touchscreen monitor (M), and the oxygen-flow control knob (K) [15,17]. In addition, the gloves (G) of the EMS crew chief were also sampled by swabbing the palm of both gloves. Selected surfaces were sampled immediately after the COVID-19 transport (T0). Decontamination SOPs were then carried out. They consisted of general clean-up, surface disinfection with sodium hypochlorite 0.1%, and a 20 min ozone saturation of the cabin. Selected surfaces were swabbed again after the decontamination of the ambulance (T1). Due to the compulsory glove change, the EMS crew chiefs’ gloves could only be sampled at T0. Sigma Virocult^®^ swabs (MWE, Corsham, England, UK) were used for sampling. The swabbed surface area was 25 × 25 cm for flat items (e.g., monitors) and consisted of the whole outer surface for non-flat objects (e.g., oxygen knobs). After sampling, the swabs were refrigerated at 4 °C and processed within 24 h.

Swabs were thoroughly vortexed for 15 s. Viral nucleic acid extraction was then performed on 140 µL of the vortexed sample with a commercial kit (QIAamp viral RNA mini kit, Qiagen, Hilden, Germany), following the manufacturer’s instructions, and yielding 60 µL of RNA extract. An internal RNA positive control (VetMAX^TM^ Xeno^TM^ IPC, Thermo Fisher Scientific, Waltham, MA, USA) was added to each sample prior to extraction, so as to assess the extraction process efficiency and the presence of PCR inhibitors. The abovementioned kit also contained a dedicated, real-time PCR assay. Molecular detection of SARS-CoV-2 RNA was performed with a previously published RT-qPCR assay, targeting gene N with a limit of detection (LOD) of 2.5 genome copies/µL [18]. Custom primers and a dual-labeled probe (5′-FAM, 3′-QSY) were purchased from Thermo Fisher Scientific (Waltham, MA, USA). Synthetic dsDNA fragments (GeneArt, Thermo Fisher, Waltham, MA, USA) were used as SARS-CoV-2 positive controls. For each PCR run, 2 positive and 2 negative controls were also included. PCR runs were performed on a StepOnePlus™ Real-Time PCR System (Applied Biosystems, Foster City, CA, USA). Positivity was qualitatively attributed to reactions with a cycle threshold (Ct) < 40 [19].

Purposely, laboratory results were shared with neither the involved EMS staff nor the decontamination technicians before sampling was completed in order to avoid performance bias due to increased awareness in either patient handling or decontamination SOPs.

## 3. Results

Overall, 11 EMS transports were sampled. Nine transports were confirmed as true COVID-19 services by SARS-CoV-2 molecular testing of the patient. One clinical swab could not be retrieved. The heart-failure patient indeed tested negative for SARS-CoV-2.

With 9 sampling points per transport, a total of 99 swabs were processed (Table 1) and 16 (16.2%) obtained a positive PCR result, thus detecting SARS-CoV-2 contamination.

In detail, SARS-CoV-2 nucleic acid was found on 4 gloves; 1 sliding-door handle; 5 touchscreen monitors; 1 oxygen knob and 5 stretcher handrails. A total of 6 ambulances out of 11 transports (54.5%) had at least one positive surface swab: all were related to a confirmed COVID-19 case. Among the 5 negative transports, 1 dealt with the heart-failure patient and 4 with confirmed COVID-19 cases.

Among the 6 positive transports, when considering the pre/post decontamination variable, the monitor resulted as the most frequently contaminated surface at T0 (83.3% of transports) although it always tested negative at T1. Three stretcher handles tested positive at T0 (50.0%), and two of them maintained positivity at T1. One sliding-door handle and 1 oxygen knob were positive at T0 (16.7% each), while all of their respective T1 swabs tested negative.

The EMS crew chiefs’ gloves resulted as contaminated in four transports (66.7%). Whenever gloves scored positive, 1–3 additional surfaces (i.e., monitor, knob, and stretcher) also resulted contaminated by SARS-CoV-2.

## 4. Discussion

The Essential Assistance Levels (LEAs) are the services that the Italian National Health Service provides to Italian citizens, free of charge or upon participation, using public funds collected through taxation. The LEAs also describe the territorial EMS network, which in Italy is mainly entrusted to the public 118 Health Emergency System. Currently, the unique European Emergency Number, 112, in which various types of distress calls can converge, is active in only about half of the country’s administrative regions.

Specific organizational choices and the programming of EMS services fall within each region’s competence to meet local peculiarities. Operating models for EMS services can thus slightly differ from region to region. Some entrust the EMS management to a specific regional agency. Elsewhere, the coordination and interlacing of distinct health agencies have been established. Finally, some Italian regions have adopted a centralized management model, which appears to correlate with a greater degree of autonomy and management efficiency.

The present study was conducted in the Veneto region (NE Italy), where the organization of EMS services is coordinated by seven Operations Centers (OCs), each located in a distinct province. Within each province, there are multiple Territorial Stations from which emergency vehicles depart in response to the received distress calls. OCs coordinate the whole sequence of activities, ensuring the transport of patients to the nearest and most appropriate hospital. OCs also assess the degree of complexity of the interventions to dispatch the most appropriate resources, using shared procedures and protocols. The degree of complexity of the intervention is defined by the dispatch system. This specific OC function encompasses the different phases of the rescue, starting from the reception of the distress call, up to the on-site arrival of the basic or advanced life-support crews.

Since the beginning of the COVID-19 pandemic, the Veneto regional health system has promoted a differentiated use of ambulances. A set number of emergency vehicles was exclusively engaged for either COVID-19-confirmed cases or PUIs. Nevertheless, should the effectiveness of decontamination be inadequate, non-infected patients and EMS crews could have been exposed to SARS-CoV-2 during the transport.

The present study aimed to assess the effectiveness of the national SOPs for the decontamination of ambulances, adopting a pre-/poststudy design, in which selected environmental surfaces were sampled both before and after selected EMS transports. Proper ambulance decontamination is known to drastically reduce the microbial contamination of surfaces touched by patients, especially those with a high viral load. Past studies have also revealed the risk of the microbial contamination of high-touch surfaces in the ambulance setting by the EMS crew, resulting in potential healthcare-associated infections (HAIs) [6]. On the one hand, the literature already supported the efficacy of decontamination SOPs for non-enveloped viruses, which are characterized by higher environmental persistence than enveloped coronaviruses [20]. However, EMS personnel have been under extreme pressure during the SARS-CoV-2 pandemic. Despite being trained to provide medical care in life-threatening circumstances, EMS crew members bore the burden of an unprecedented healthcare crisis that could have led to a decline in safety procedures. Following the transport of a COVID-19-positive patient, the risk of contaminating the ambulance could be remarkable, thus implying a considerable risk of infection for other patients and the EMS crew.

In the present study a non-COVID-19 EMS transport was considered (i.e., Transport 0 as a “blank”: a heart-failure patient with a negative molecular swab) for analytical reference; no positive environmental sample was detected at either T0 or T1. The ten remaining transports concerned COVID-19 confirmed cases, except Transport 2, since investigators could not retrieve the result of the SARS-CoV-2 testing. Four out of the ten COVID-19 transports (i.e., 3, 6, 8, and 10) were characterized by negative environmental samples, both at T0 and T1, whereas 6 ambulances had at least 1 positive environmental swab, supporting the SARS-CoV-2 contamination of the vehicle’s cabin. The rational use and removal of personal protective equipment (PPE) for coronavirus disease, both for healthcare workers and for transported patients, are eligible as the main factors preventing the environmental contamination of the ambulance setting [13] and MTAIs [5]. The correct performance of decontamination SOPs is also fundamental in minimizing the accidental, post-transport contamination of cabin surfaces.

At T0, Transport 1 had three positive samples (the monitor, door handle, and oxygen knob), plus the gloves of the crew chief. However, no positive swabs were found at T1, suggesting the correct accomplishment of the decontamination SOPs. Transport 2 and Transport 4 both shared a similar pattern: the gloves, monitor, and stretcher were positive at T0, and the positivity of the latter was also maintained at T1, possibly due to the stretcher being dismounted from the ambulance and thus bypassing the correct decontamination SOPs. The remaining three EMS transports had at least one positive sample at T0, but none at T1: Transport 5 had positive gloves and monitor; Transports 7 and 9 showed positivity for the stretcher and monitor, respectively.

Switching to a sampling-point perspective, it emerged that SARS-CoV-2-positive gloves always resulted in the contamination of the ambulance cabin. Overall, this finding reprises the critical role of correct hand hygiene and PPE use. Some pre-pandemic studies have already discussed how EMS personnel could mediate the transmission of microbial pathogens if hand hygiene is not performed correctly [21]. In addition, a recent study centered on a simulation-based assessment of ambulance and crew contamination during the COVID-19 pandemic identified bilateral hands (i.e., gloves) as one of the most frequently contaminated areas before the removal of PPE [13].

Only one sliding-door handle resulted as contaminated, marking this sampling point as at low contamination risk. Consistently, the opening and closing of the vehicle’s sliding door is usually performed by the driver, who has no direct interaction with the transported patient. Similarly, the oxygen knob seemed to be a low-risk sampling site, with only one positive swab at T0. However, since we could not retrieve any information on oxygen usage during each transport, correlating to the severity of the transported patient, the actual contamination risk for the knob remains uncertain.

The multiparameter monitors (positivity at T0 in 5 transports) and the stretcher handrail (positivity at T0 in 3 transports) emerged as the surfaces with the highest probability of contamination, especially if the gloves of the crew chief were infected, too. However, the monitor decontamination always resulted successfully, whereas two out of three EMS transports also maintained positivity for the stretcher at T1. On the one hand, the flat and smooth monitor surface can be easily decontaminated. On the other hand, the haste of EMS operations during patient dismount, together with the juxtaposed actions of multiple healthcare workers, probably interfered with the correct stretcher decontamination.

Thus far, the present study has the following two limitations: First, it would have been preferable to conduct field investigations on a larger number of EMS transports and, especially, to recruit more than two EMS bases/crews, so as to lessen the effect of possible operator bias. By enlarging the sampling activities, we could also strengthen our inferences on possible faults of decontamination SOPs over a wider territory; second, the severity of each patient’s health condition during the EMS transport was not available, and we could not evaluate its influence on surface contamination nor on the accuracy of the decontamination procedures (e.g., patient with severe cough, critical condition, etc.).

Research perspectives, strongly supported by the authors, should therefore include further field research to increase pragmatic knowledge on the proper meeting of hygienic quality requirements for the particular indoor setting of ambulances and EMS vehicles. In particular, the definition of specific methods to perform the assessment of microbiological air quality in the ambulance cabin is needed.

## 5. Conclusions

Since the early stages of the COVID-19 pandemic, effective decontamination SOPs have played a crucial role in preventing SARS-CoV-2 MTAIs on ambulances. Moreover, the proven persistence of SARS-CoV-2 on surfaces in hospital settings has highlighted the importance of hygiene fundamentals to control the virus’s environmental transmission. The present study suggested how theoretical efficiency (i.e., the extent to which they minimize the risk of infection, under ideal and controlled circumstances) of decontamination SOPs can be considered safe. Nevertheless, their effectiveness (i.e., their performance in the real EMS context) seemed to be easily flawed by the cursory behaviors of the EMS crews, such as imperfect hand hygiene and hasty stretcher management.

Improving compliance with correct SOPs appears to be the most efficient and achievable approach. The dedicated and continuous training of EMS personnel and the consolidation of critical decontamination steps should be supported and promoted by local EMS bases under the guidance of the regional health authorities. Moreover, additional environmental evaluations (e.g., cabin air quality assessments) and scheduled monitoring programs should be encouraged, as complimentary research investigation to further elucidate possible discrepancies between the theoretical efficacy and the pragmatic effectiveness of decontamination SOPs.

## Figures and Tables

**Table 1:** SARS-CoV-2 contamination of high-touch surfaces in ambulances. Ambulance contamination was present (+) if at least one surface swab tested positive for SARS-CoV-2. The table also reports the results of each patient’s SARS-CoV-2 test (+/−) upon hospital admission. Results of the environmental swabs are presented for each sampling site, both pre- (T0) and post- (T1) decontamination procedures.

EMS ID	Ambulance Contamination	Patient SARS-CoV-2 Testing	T0-G	T0-H	T0-M	T0-K	T0-S	T1-H	T1-M	T1-K	T1-S
0 *	−	−	−	−	−	−	−	−	−	−	−
1	+	+	+	+	+	+	−	−	−	−	−
2	+	n.a.	+	−	+	−	+	−	−	−	+
3	−	+	−	−	−	−	−	−	−	−	−
4	+	+	+	−	+	−	+	−	−	−	+
5	+	+	+	−	+	−	−	−	−	−	−
6	−	+	−	−	−	−	−	−	−	−	−
7	+	+	−	−	−	−	+	−	−	−	−
8	−	+	−	−	−	−	−	−	−	−	−
9	+	+	−	−	+	−	−	−	−	−	−
10	−	+	−	−	−	−	−	−	−	−	−

* heart-failure patient; n.a.—not assessed; T0—predecontamination; T1—postdecontamination; G—gloves; H—door handle; M—monitor; K—oxygen knob; S—stretcher handle.
